# Red Blood Cell Proteasome in Beta-Thalassemia Trait: Topology of Activity and Networking in Blood Bank Conditions

**DOI:** 10.3390/membranes11090716

**Published:** 2021-09-17

**Authors:** Alkmini T. Anastasiadi, Vassilis L. Tzounakas, Vasiliki-Zoi Arvaniti, Monika Dzieciatkowska, Konstantinos Stamoulis, Marilena E. Lekka, Issidora S. Papassideri, Angelo D’Alessandro, Anastasios G. Kriebardis, Marianna H. Antonelou

**Affiliations:** 1 Department of Biology, School of Science, National and Kapodistrian University of Athens (NKUA), 15784 Athens, Greece; alkanast@biol.uoa.gr (A.T.A.); tzounak@biol.uoa.gr (V.L.T.); vazoarvaniti@gmail.com (V.-Z.A.); ipapasid@biol.uoa.gr (I.S.P.); 2 Department of Biochemistry and Molecular Genetics, School of Medicine, Anschutz Medical Campus, University of Colorado, Aurora, CO 80045, USA; monika.dzieciatkowska@ucdenver.edu (M.D.); angelo.dalessandro@ucdenver.edu (A.D.); 3 Hellenic National Blood Transfusion Centre, Acharnes, 13677 Athens, Greece; kostas.stamoulis@gmail.com; 4 Laboratory of Biochemistry, Department of Chemistry, University of Ioannina, 45110 Ioannina, Greece; 5 Laboratory of Reliability and Quality Control in Laboratory Hematology (HemQcR), Department of Biomedical Sciences, School of Health & Welfare Sciences, University of West Attica (UniWA), 12243 Egaleo, Greece; akrieb@uniwa.gr

**Keywords:** red blood cell, proteostasis, proteasome, activity, regulation, membrane localization, interactome, storage, beta thalassemia trait

## Abstract

Proteasomes are multi-catalytic complexes with important roles in protein control. Their activity in stored red blood cells (RBCs) is affected by both storage time and the donor’s characteristics. However, apart from their abundancy in the membrane proteome, not much is known about their topology, activity, and networking during the storage of RBCs from beta-thalassemia trait donors (βThal^+^). For this purpose, RBC units from fourteen βThal^+^ donors were fractionated and studied for proteasome activity distribution and interactome through fluorometric and correlation analyses against units of sex- and aged-matched controls. In all the samples examined, we observed a time-dependent translocation and/or activation of the proteasome in the membrane and a tight connection of activity with the oxidative burden of cells. Proteasomes were more active in the βThal^+^ membranes and supernatants, while the early storage networking of 20S core particles and activities showed a higher degree of connectivity with chaperones, calpains, and peroxiredoxins, which were nonetheless present in all interactomes. Moreover, the βThal^+^ interactomes were specially enriched in kinases, metabolic enzymes, and proteins differentially expressed in βThal^+^ membrane, including arginase-1, piezo-1, and phospholipid scramblase. Overall, it seems that βThal^+^ erythrocytes maintain a considerable “proteo-vigilance” during storage, which is closely connected to their distinct antioxidant dynamics and membrane protein profile.

## 1. Introduction

Proteasomes are supramolecular, multi-catalytic complexes tasked with the degradation of aberrant and damaged proteins, which is needed to ensure cell homeostasis [1]. As such, their proteolytic core, the 20S complex, is equipped with three proteolytic activities: caspase (CASP)-like, trypsin (TR)-like, and chymotrypsin (CH)-like, the active centers of which are located at the β1, β2, and β5 subunits of the catalytic chamber, respectively. Misfolded proteins are transferred in the 20S cylinder after being selected by 19S regulatory complexes that recognize ubiquitinated molecules [2]. Of note, while the function of 26S holoenzymes is ATP- and ubiquitin-dependent, the 20S proteasome requires neither [3,4]. The importance of these molecules becomes apparent through their involvement in a number of processes, from post-translational processing [5] to regulation of cell cycle [6], signal transduction, and response to oxidative stress [7,8]. Deregulation of the proteasome results in defects in both proliferation or apoptotic processes and consequently the development of pathologies [9].

In red blood cells (RBCs), particularly, proteasomes are of great interest given the finite nature of the RBCs’ proteome. These multi-catalytic machines (“near-organelles”) are retained in mature erythrocytes, while the typical organelles are expelled during late erythropoiesis. Their presence and functionality in the RBC cytosol and membrane has been confirmed by several studies [10,11], while an intriguing relative excess of the 20S core over the 26S holoenzyme has been reported at ≈20×-fold. Given that oxidative stress is a significant challenge for erythrocytes, this abundance could be attributed to the fact that 20S proteasomes are both more efficient in degrading oxidized proteins [12] but also more resilient against oxidative damage themselves [13]. RBC’s proteasomal content and activity are responsive to changes in physiological conditions or to diseases, including anemia [14,15]. Proteasomal subunits have been found upregulated in the RBC cytosol of sickle cell disease patients [16].

In the context of the proteasome’s presence and role during RBC storage under blood bank conditions, little is known [17]. However, recent findings show that proteasomal activities demonstrate a decrease in cytosol and an increase in membrane during storage, suggesting a recruitment of proteasomes along with other components of the “repair or destroy” system to the membrane [18]. Moreover, proteasomal activities were detected in extracellular vesicles [17,18] and the unit’s supernatant [19], hinting at the release of complexes during late storage. By studying the donor variation effect upon the storage profile of RBCs, the genetically discrete G6PD-deficient RBCs have been shown to exhibit higher levels of proteasomal activity and release during storage [18], whereas membranes from post-menopausal women demonstrate a trend for slightly lower activity levels [20] compared to controls and pre-menopausal women, respectively.

RBCs from heterozygotes for beta-thalassemia (βThal^+^) face oxidative challenges due to their genetic background [21]. Nevertheless, during storage—a period strongly associated with oxidative and proteotoxic stresses—they seem to employ a number of protective mechanisms to mitigate this predisposition. In our previous works, we observed lower reactive oxygen species (ROS) levels in late storage and a shift of the metabolism toward the Pentose Phosphate Pathway (PPP), suggesting a higher antioxidant potential compared to controls [22]. Furthermore, a recent comparison of the proteome of βThal^+^ and control membranes revealed enrichment of the former in a number of “repair or destroy” molecules during storage, such as chaperone HSP70 and proteasomal subunits, among others [23]. The effectiveness of these compiled protective pathways is also supported by reports of lower levels of protein carbonylation, lipid peroxidation, and other oxidative stress indices, such as the metabolite allantoin during storage in this group [22]. Altogether, (a) the effect of genetic background upon proteasome activity during storage, (b) the multiple hints towards a superior redox and proteostatic profile in βThal^+^ stored RBCs, and especially, the differential binding of proteasome subunits in their membrane, and (c) the importance of protein control for the structural and functional integrity of erythrocytes during the stressful period of storage (and probably, later on, in the recipient of transfusion) motivated us to examine the proteasome activity, sub-cellular/extracellular distribution, and networking in stored RBCs donated from healthy, eligible βThal^+^ volunteers, to further evaluate their storability compared to control.

## 2. Materials and Methods

### 2.1. Biological Samples

Freshly drawn blood in citrate vacutainers and packed RBCs in citrate-phosphate-dextrose (CPD)/saline-adenine-glucose-mannitol (SAGM) leukoreduced units were donated by twenty-eight (14 βThal^+^ and 14 control) healthy volunteers, as previously reported [22]. The blood units were stored for 42 days at 4 °C and were sampled every week under aseptic conditions. The beta-thalassemia trait was confirmed by both Hb electrophoresis and molecular identification of mutations. The study was approved by the Ethics Committee of the Department of Biology, School of Science, NKUA. Investigations were carried out upon donor consent in accordance with the principles of the Declaration of Helsinki.

### 2.2. Sample Fractionation

RBCs and plasma/supernatant were separated by centrifugation at 1000× *g* for 10 min at 4 °C. Then, a volume of RBCs was used for fractionation into membrane and cytosol fractions by centrifugation (19,000× *g*) following osmotic lysis in hypotonic sodium phosphate buffer (pH 8.0) containing 0.3 mM phenyl-methyl-sulfonyl fluoride as a protease inhibitor at 4 °C. Cytosol fractions were stored for further analysis, and the pellets of RBC membranes, along with the submembrane cytoskeleton, were repeatedly washed under the same conditions to remove the excess of bound hemoglobin. Extracellular vesicles (EVs, mostly microvesicles) were isolated by the cell-free supernatant of the RBC units by centrifugation at 37,000× *g* (n = 5 per group), post filtering through sterile 0.8 µm nitrocellulose filters (Millipore, Carrigtwohill, County Cork, Ireland), at late storage (day 42) [24]. Proteasome activity was measured in all fractions.

### 2.3. Proteasome Activities

Proteasome activity in cytosol, membrane, plasma/supernatant, and EV samples was determined by using fluorogenic substrates in a fluorometry approach [18,25]. Briefly, 120–200 µg of protein samples in 20 mmol/L Tris-HCl (pH 7.5 for chymotrypsin-like or 8.0 for the other two activities) were incubated with the peptide substrates Suc-Leu-Leu-Val-Tyr-aminomethylcoumarin (AMC) (for chymotrypsin-like activity), z-Leu-Leu-Glu-AMC (for caspase-like activity), and Boc-Leu-Arg-Arg-AMC (for trypsin-like activity) for 1.30 h (chymotrypsin-like) or 3 h (caspase-like and trypsin-like) at 37 °C in the dark. The relative fluorescence units (excitation filter: 365 nm; emission filter: 460-VersaFluor, BIO-RAD Hercules, CA, USA) were thereafter normalized to protein concentration (Bradford protein assay, Bio-Rad, Hercules, CA, USA). The experiments were also performed in the presence of inhibitors (10–20 μΜ bortezomib for the chymotrypsin and caspase-like activities, 200 μΜ MG-132 for the chymotrypsin-like activity, and 100 μM lactacystin for the tryspin-like activity) to discriminate between the generic proteolytic activity and that of proteasome machinery. All proteasome data reported are the final values resulting from the subtraction of the unspecific portion of activity measured by the use of inhibitors (92–96% inhibition of proteasome activities). All substrates and inhibitors were procured from Enzo Life Sciences (New York, NY, USA).

### 2.4. Physiological and Proteomic Parameters

The hematological (MCV, MCH etc.), physiological (e.g., hemolysis, osmotic fragility, antioxidant capacity, reactive oxygen species (ROS)), and protein (e.g., carbonylation) parameters used for the correlation analyses (scatterplots and biological networks) originate from specific experiments that were performed in the same cohorts of donors as thoroughly analyzed and reported in recent publications related to this project [22,23]. Intracellular levels of reactive oxygen species (ROS) were assayed with the help of the redox-sensitive and membrane-permeable probe 5-(and-6)-chloromethyl-2′,7′-dichloro-dihydro-fluorescein diacetate, acetyl ester (CM-H_2_DCFDA, Invitrogen, Molecular Probes, Eugene, OR, USA), as extensively described before [26]. ROS accumulation was also determined following stimulation of the cells with the oxidants tert-butyl-hydroperoxide (tBHP), diamide, and phenylhydrazine. Proteomic analysis was performed in 12 membrane samples of each group in early (day 7) and late (day 42) storage, as well as in 5 EV samples of each group, through a FASP digestion prior to analysis via nano-UHPLC-MS/MS (Evosep One system coupled to timsTOF Pro mass spectrometer-Bruker Daltonics, Bremen, Germany), as extensively described before [23].

### 2.5. Statistical and Network Analysis

All experiments were performed in triplicate. Statistical analysis was performed by using the statistical package SPSS Version 22.0 (IBM Hellas, Athens, Greece, administered by NKUA). Repeated measures ANOVA with Bonferroni-like adjustment for multiple comparisons was used for the evaluation of time-course and between groups differences. Correlation between physiological and proteomic parameters of stored RBCs was assessed with Pearson’s or Spearman’s tests after testing for normal distribution and the presence of outliers (Shapiro-Wilk test and detrended normal Q–Q plots). The R values were used for the construction of biological networks (Cytoscape 3.7.2, San Diego, CA, USA). Significance was accepted at *p* < 0.05 or *p* < 0.01 (in the case of network analysis).

## 3. Results

### 3.1. Time-Course Evaluation of Proteasome Activity during RBC Storage

The three catalytic activities of the proteasome presented cytosol-, membrane-, and supernatant-specific time-course profiles in the total set of donor samples (Table 1). In the cytosol, there was a decrease in all activities in middle storage when compared to day 7, which further declined later (CH-like and CASP-like activities). An opposite pattern was observed in the membrane: both CASP-like and TR-like activities reached maximum values in the middle of the storage period (peaked at day 21) before a subsequent reduction to early storage levels. Likewise, CH-like activity presented an upward trend from early to middle storage before a statistically significant decrease toward day 42. Moving out of the cell, the activities of the proteasome in the supernatant were characterized by a steady trend for increase during storage, which reached statistical significance either in the middle (CASP-like) or the later periods of it (TR-like and CH-like) (Table 1).

### 3.2. Proteasome Activity in βThal^+^ versus Control RBCs

**Figure 1:**
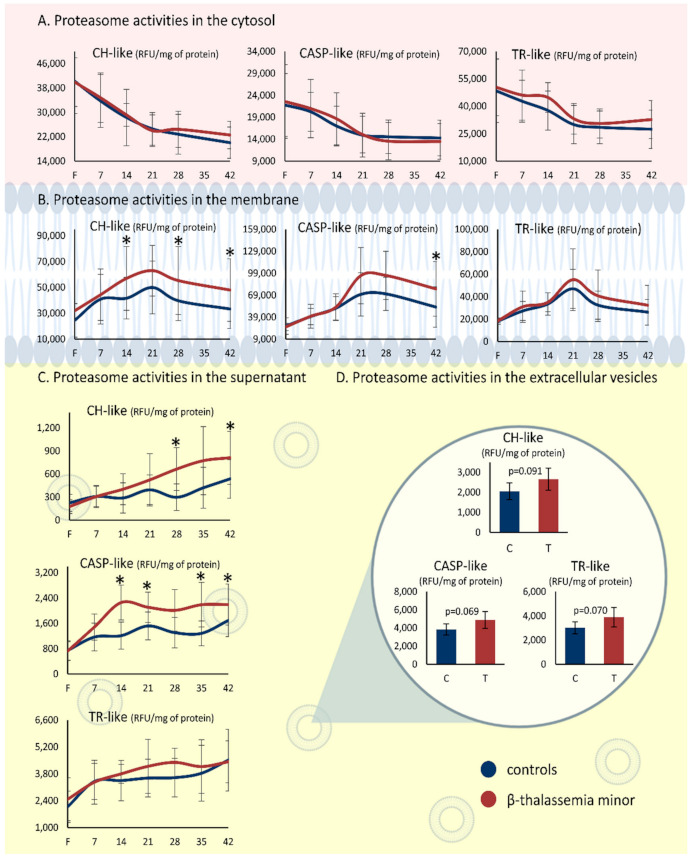
Proteasome activities in red blood cells of beta-thalassemia minor and control donors. Chymotrypsin (CH)-, caspase (CASP)-, and trypsin (TR)-like activities in (**A**) RBC cytosol, (**B**) RBC membrane, (**C**) RBC units’ supernatant, and (**D**) RBC-derived extracellular vesicles on day 42. Horizontal axis: days of storage; F: freshly drawn blood; (*) *p* < 0.05 between βThal^+^ and controls. Created with BioRender.com.

Despite having a similar variance profile over storage time inside and outside RBCs, the levels of the proteasome activity differed between the two donor groups in both membrane and supernatant fractions. While the βThal^+^ RBCs had control levels of proteasome activities in the cytosol (Figure 1A), they showed a marginal trend for higher CH-like and CASP-like values in the membrane from mid-storage onwards (e.g., CASP-like day 21: 95,742 ± 38,444 vs. 70,122 ± 29,524 RFU/mg of protein, βThal^+^ vs. control, *p* = 0.052) (Figure 1B). Of note, while in the overall sum of the samples, CH-like activity did not present a statistical zenith in the membrane on day 21 of storage (as observed in the other two activities; see Table 1), when the analysis was performed for the two groups separately, this pattern was revealed in the βThal^+^ but not in the control membrane samples (day 7 vs. day 21, *p* = 0.001). Apart from the membrane, the CH-like and CASP-like activities were higher in the supernatant of the βThal^+^ units as well, starting from the second (CASP-like) or the fourth week of storage (CH-like) (Figure 1C). Finally, the proteasome activities presented statistical trends for elevated levels in the βThal^+^ vs. control EVs isolated by the supernatant on day 42 of storage (Figure 1D).

### 3.3. Correlations between Proteasome Activities and Reactive Oxygen Species in βThal^+^ and Control RBCs

**Figure 2:**
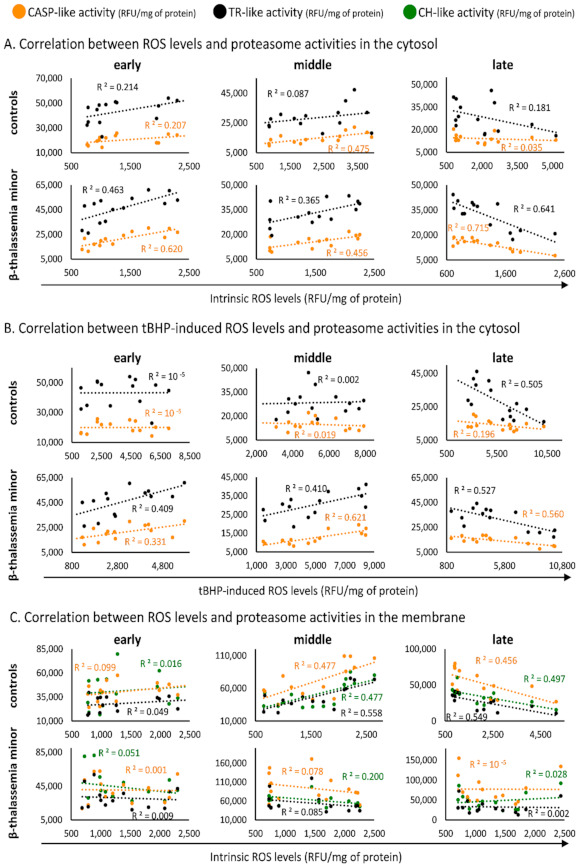
Correlation analysis between reactive oxygen species (ROS) generation and proteasome activities. Indicative scatter plots between (**A**) intrinsic ROS levels and cytosolic proteasome activities, (**B**) tert-butyl-hydroperoxide (tBHP)-induced ROS levels and cytosolic proteasome activities, and (**C**) intrinsic ROS levels and membrane proteasome activities. CASP: caspase, TR: trypsin, CH: chymotrypsin. R ^2^ values > 0.284 are statistically significant at *p* < 0.05.

Since the proteasome activities in RBCs are responsive to oxidative stress, we next sought to look for statistically significant correlations between them and ROS accumulation in βThal^+^ and control RBCs as a function of topology and storage time. Interestingly, the cytosolic CASP-like and TR-like activities correlated positively with the intrinsic ROS levels during early and middle storage but negatively at late storage in the βThal^+^ (but not in control) samples (Figure 2A). 

The same pattern was observed for ROS accumulation in stored RBCs challenged with tBHP (Figure 2B) or diamide (e.g., TR-like middle storage, βThal^+^: R^2^ = 0.635, *p* = 0.013; control: R^2^ = 0.110, *p* > 0.05). Notably, only the CASP-like cytosolic activity correlated (positively or negatively in early/middle or late storage, respectively) with ROS levels following treatment of RBCs with the Hb-targeting phenylhydrazine, and again, this correlation was only observed in βThal^+^ RBCs (e.g., middle storage, βThal^+^: R^2^ = 0.574, *p* = 0.001; control: R^2^ = 0.024, *p* > 0.05). Regarding the proteasome activities in the membrane of βThal^+^ RBCs, they showed no correlations with the ROS levels (throughout the storage period) in contrast to controls, which exhibited strong positive or negative correlations during middle or late storage, respectively (Figure 2C).

### 3.4. Biological Networks

#### 3.4.1. Biological Networking of Proteomic and Physiological Parameters of RBCs

**Figure 3:**
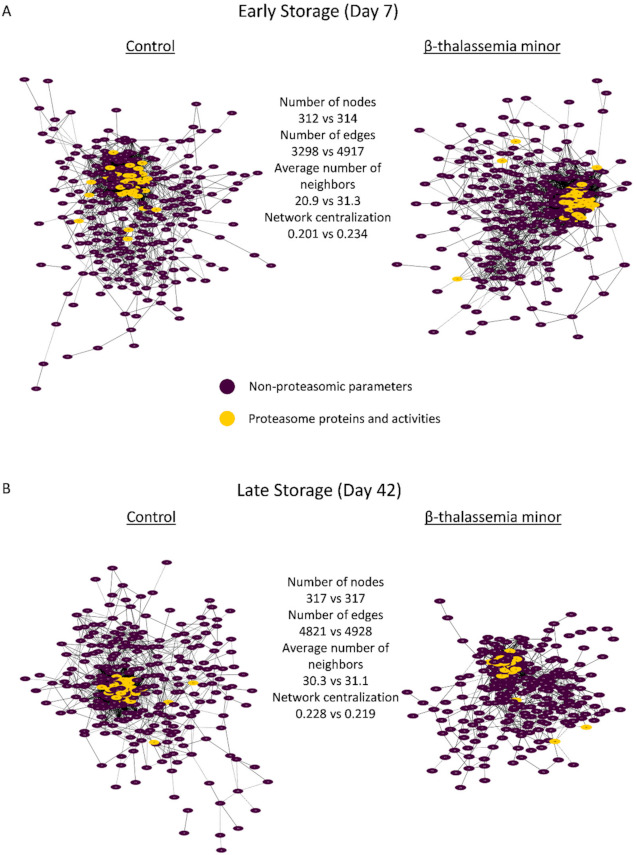
Network analysis of proteomic and physiological parameters in beta-thalassemia minor and control stored red blood cells during early (**A**) or late (**B**) storage. The connections represent statistically significant correlations (*p* < 0.01) between RBC proteins found on the membrane/cytoskeleton and physiological parameters. Solid lines: positive correlation; dashed lines: negative correlation. Node numbers correspond to the definitions provided in Table S1.

Network analysis was performed for the topological visualization of the statistically significant correlations between a great variety (>300 nodes) of omics (RBC membrane proteome) and physiological (e.g., cellular fragility, redox equilibrium) parameters of the βThal^+^ and control RBC units [22,23], including the proteasome (components and activities). This approach helps to elucidate the possible associations between parameters of stored RBCs that may fuel future more targeted research concerning the regulation of proteasome activity, interactions, recruitment to the membrane, and release during storage. Those complex biological interactomes revealed the centrality and compactness of proteasome-related factors in both groups during the early and late periods of storage (Figure 3). Despite having a similar number of nodes and degree of centralization, the βThal^+^ network contained almost 1.5-fold the connections of the control network in early storage, as evidenced by the average number of neighbors per node. However, the number of connections of the βThal^+^ network did not increase in late storage as opposed to the control, leading to quantitatively equal networks at the end of the storage period.

#### 3.4.2. Biological Networking of 20S Proteasome

**Figure 4:**
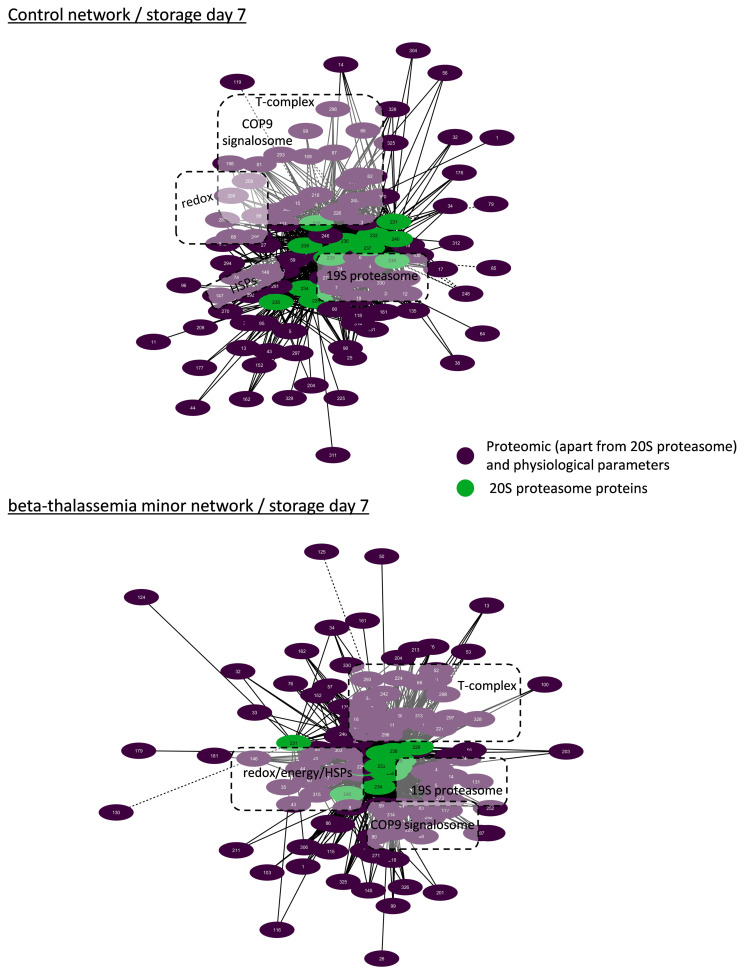
Network analysis for core 20S proteasome proteins in beta-thalassemia minor and control stored red blood cells during early storage. Interactomes showing statistically significant correlations (*p* < 0.01) between RBC parameters and 20S proteasome proteins. Solid lines: positive correlation; dashed lines: negative correlation. Node numbers correspond to the definitions provided in Table S1.

**Figure 5:**
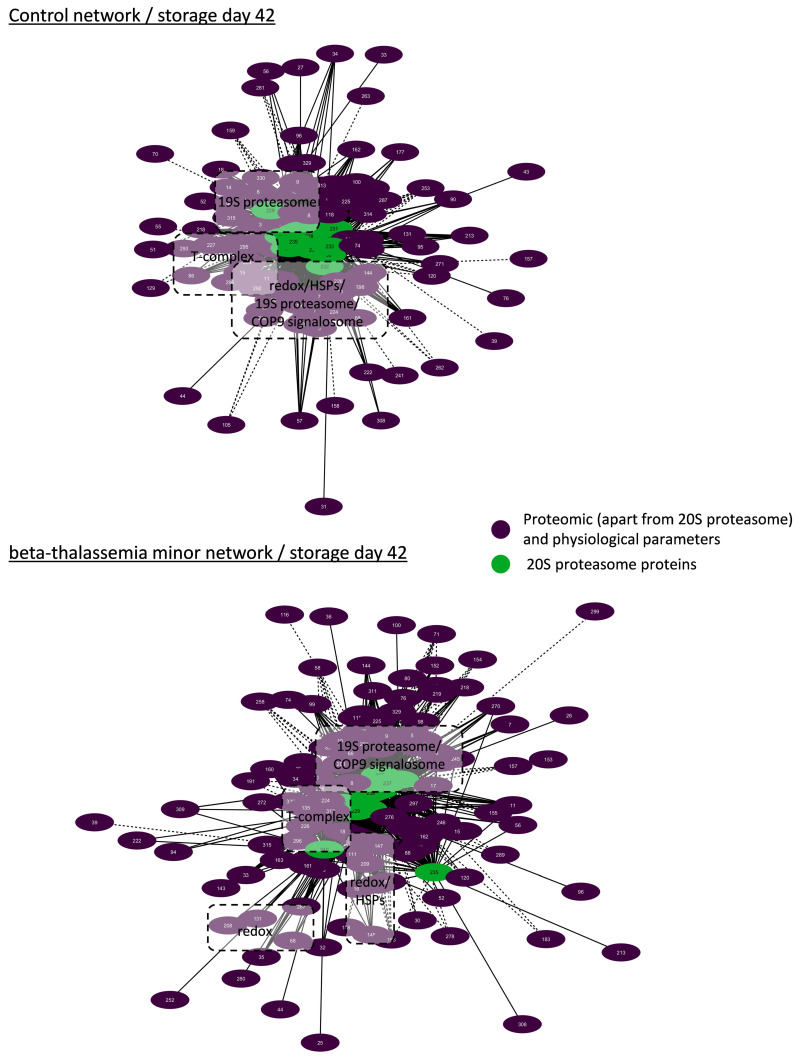
Network analysis for core 20S proteasome proteins in beta-thalassemia minor and control stored red blood cells during late storage. Interactomes showing statistically significant correlations (*p* < 0.01) between RBC parameters and 20S proteasome proteins. Solid lines: positive correlation; dashed lines: negative correlation. Node numbers correspond to the definitions provided in Table S1.

Due to the great excess and the critical role of the 20S core proteasome in RBCs, we then focused on the core 20S interactomes in βThal^+^ and control RBCs in early (Figure 4) and late (Figure 5) storage. Those subnetworks represented as much as 25% of the parental ones. In both donor groups, the membrane-bound 20S proteasome subunits presented positive correlations with: (a) the proteasome activities, (b) several regulatory subunits of the 19S particles, proteasome partners (e.g., ECPAS), and ubiquitin-related proteins (e.g., E3 ubiquitin ligase complexes, COP9 signalosome), (c) molecular chaperones (heat shock proteins and components of the chaperonin-containing T-complex), (d) lipid biosynthesis and metabolism (e.g., fatty acid synthase), (e) calcium-dependent proteins (e.g., calpain and calpastatin), (f) energy and redox homeostasis (peroxiredoxins, catalase etc.), (g) proteins involved in nucleoside metabolism (e.g., GMP reductase, ribose-phosphate pyrophosphokinase 1), as well as (h) arginine methyltransferase (Figure 4 and Figure 5). The far fewer inverse correlations were principally found in late storage and concerned structural proteins of the membrane (e.g., glycophorins), raft residents (e.g., CD44, flotillins), small GTPases, and immunoglobins (Figure 5).

As in the case of the total networks (Figure 3), the βThal^+^ network of the 20S core proteasome started with significantly more connections when compared to the control (Figure 4) and ended up with a similar connectivity profile at late storage (Figure 5). Indeed, in early storage, the 20S core proteasome subunits were far more interconnected with chaperones (141 vs. 85 connections), calpains (19 vs. 3 connections), energy metabolism components, and peroxiredoxins (26 vs. 12 connections) in βThal^+^ compared to control (Figure 4), which was a difference that was either lost or reversed in late storage (e.g., peroxiredoxins connections: 14 vs. 25 in βThal^+^ vs. control) (Figure 5). In the last day of storage, the βThal^+^ 20S proteasome exhibited a higher degree of negative correlations with immunoglobulins (21 vs. 7 connections in control) and with a different array of key structural or raft membrane components (flotillin-1 in both groups; CD59, piezo-1, calpain, and ICAM-4 in βThal^+^; glucose transporter and RhCE in control).

Concerning the EV fractions, alpha-5 was the only 20S proteasome subunit with high connectivity (n = 6) in both groups. In βThal^+^, its levels positively correlated with protein methyltransferase PCMT1 and alpha synuclein—a typical load of extracellular vesicles—and negatively with immunoglobins and prothrombin. In control EVs, it exhibited positive correlations with the vesicular superoxide dismutase but inverse correlations with the raft-located acetylcholinesterase.

#### 3.4.3. Biological Networks of Proteasome Activities

**Figure 6:**
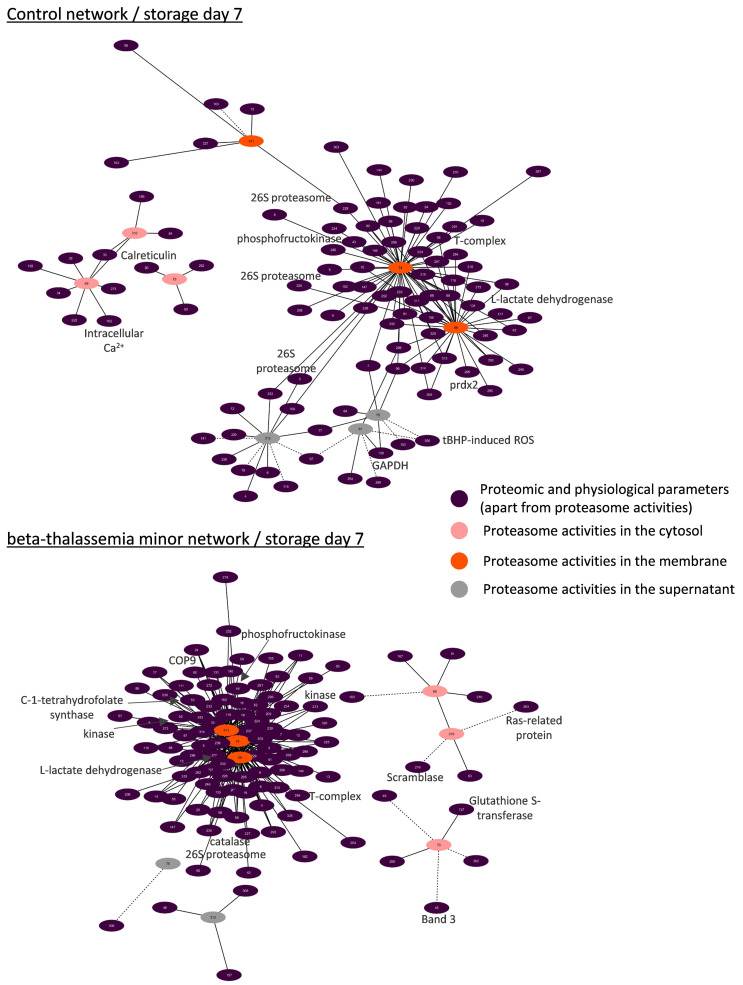
Network analysis for proteasomic activities in beta-thalassemia minor and control stored red blood cells during early storage. Interactomes showing statistically significant correlations (*p* < 0.01) between RBC parameters and proteasome activities. Solid lines: positive correlation; dashed lines: negative correlation. Node numbers correspond to the definitions provided in Table S1. Prdx2: peroxiredoxin-2; tBHP: tert-butyl-hydroperoxide.

**Figure 7:**
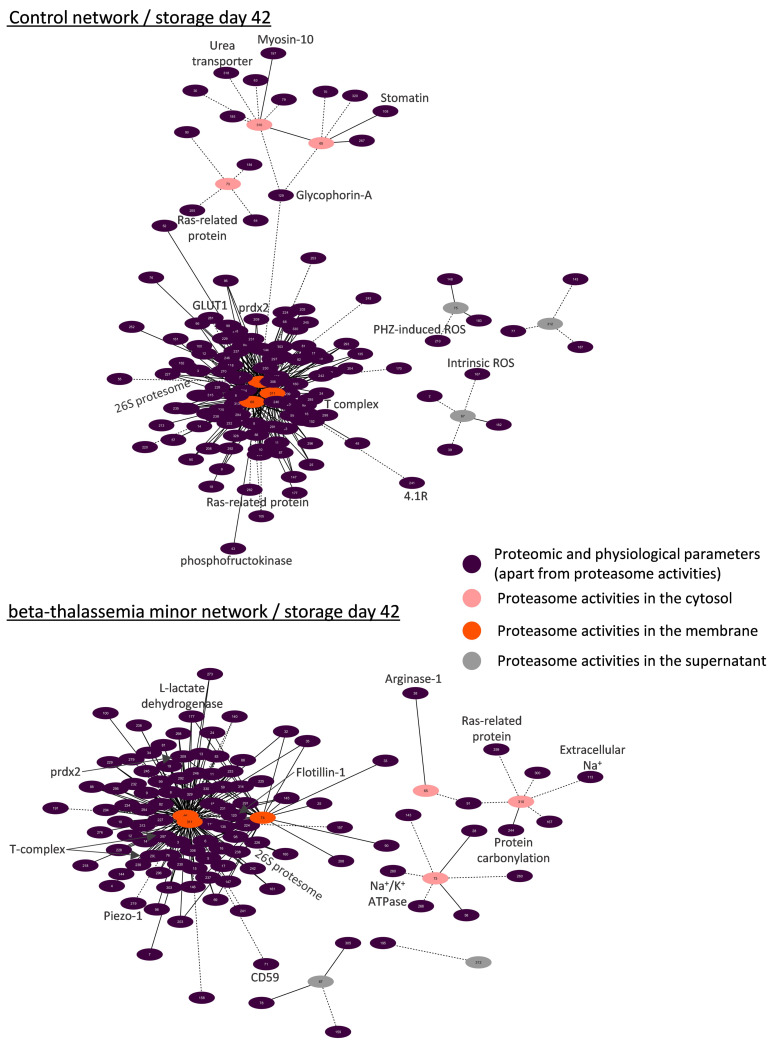
Network analysis for proteasomic activities in beta-thalassemia minor and control stored red blood cells during late storage. Interactomes showing statistically significant correlations (*p* < 0.01) between RBC parameters and proteasome activities. Solid lines: positive correlation; dashed lines: negative correlation. Node numbers correspond to the definitions provided in Table S1. Prdx2: peroxiredoxin-2; GLUT1: glucose transporter-1; PHZ: phenylhydrazine.

In a second focal plane, we visualized the interactomes of proteasome activities. Those subnetworks revealed an interesting spatial pattern in both donor groups: most of the connections (>70–90%) referred to the proteasome activities in the membrane, followed by those in the cytosol (≈6%) and the supernatant (<3%) (Figure 6 and Figure 7). Only in the early control interactome, there was significant connectivity to proteasome activities of the supernatant (20% of total) and the cytosol (10% of total) (Figure 6).

In both groups, the membrane levels of proteasome activities exhibited positive correlations with components of the 20S and 19S particles, the ubiquitin–conjugation pathway, several molecular chaperones, calpain/calpastatin, peroxiredoxins, catalase, and arginine N-methyltransferase. Interestingly, in all networks, there were also positive correlations with the fatty acid synthase (Figure 6 and Figure 7). Metabolic enzymes were more evident in the βThal^+^ networks, along with an array of kinases. On the other side, the membrane activity of the proteasome showed negative correlations with C-1-tetrahydrofolate synthase in βThal^+^ during early storage (Figure 6) and with several membrane proteins in both βThal^+^ (CD59, 4.1R protein, flotillin-1, Piezo-1) and control (4.1R protein, glucose transporter, calnexin) interactomes in the end of it. Lipid raft proteins and immunoglobins were only present in the βThal^+^ network of day 42 (Figure 7).

Apart from the correlations with intracellular ROS thoroughly analyzed in this study (Figure 2), the levels of proteasome activities in the βThal^+^ cytosol were positively connected to additional redox-related variables (e.g., glutathione S-transferase, protein carbonylation; see Figure 6 and Figure 7). Of note, some cellular and membrane protein features of βThal^+^ stored RBCs [22,23] were also present in their activity interactomes in contrast to controls: band 3, phospholipid scramblase-1, osmotic fragility (Figure 6), calpain, arginase-1, RHAG, ATPase Na^+^/K^+^ (Figure 7). On the other hand, the control networks of cytosolic activities were dominated by calcium-related (cytosolic calcium concentration, calreticulin) and internal or membrane-associated components (e.g., day 42: positive with stomatin and myosin; negative with glycophorin A, CD44), the cytosolic concentration of Hb, and several enzymes. Small GTPases were inversely connected to the cytosolic activities in both groups.

Even though the extracellular levels of the proteasome activity were significantly lower compared to those of the RBCs, they showed correlations with both membrane and cytosolic parameters, especially in the control group at early storage. Extracellular activities displayed negative association with intracellular ROS levels in the beginning (βThal^+^) or throughout storage (controls). In the group of heterozygotes, extracellular proteasome activity was further associated with skeletal components positively at early storage (Figure 6) but negatively at the later period of it (Figure 7). Some interesting correlations arose in the control networks: There were positive connections with the levels of membrane-bound 20S and 19S subunits, ubiquitin–protein ligases, chaperones, glycolytic enzymes, kinases, and their regulators during early storage, as well as with the hematocrit of the unit, the mean volume, and the osmotic fragility of stored RBCs at the end of it. On the contrary, inverse correlations with complement receptor-1, membrane-bound immunoglobins (day 7), heat shock proteins, and lipid peroxidation (day 42) were also observed (Figure 6 and Figure 7).

## 4. Discussion

Recent studies of our team revealed that RBCs from βThal^+^ donors possess (a) superior storage lesion profile in terms of hemolysis (storage, osmotic, mechanical, oxidative) and secondary quality metrics such as removal signaling, (b) resistance to the storage-related oxidative stress in comparison to the average RBCs [22], and (c) unique proteomic signatures at the membrane, which is consistent with their metabolic and physiological features [23]. The present study expands our work in βThal^+^ donors by reporting for the first-time different levels of activity and networking of the proteasome in the freshly drawn and stored βThal^+^ RBCs. These findings are in line with the recently observed abundancy of proteasome proteins in βThal^+^ membranes [23] as well as with the effects of the donor’s genetic background and storage time upon proteasome topology and activity [18].

### 4.1. The Discrete Spatiotemporal Proteasome Activity Profile in Stored RBCs

Based on our results, storage is associated with a tendency for decreasing proteasome activity in the cytosol and a temporal increase in the membrane activity levels around day 21, implying the translocation of active proteasome subunits to the membrane or activation of inactive membrane proteasomes in situ in both donor groups. Similar distributions were detected in stored RBCs from G6PD-deficient donors [18], establishing a storage-driven spatiotemporal proteasome activity profile in RBCs. It is known that storage in the cold promotes ROS generation, oxidative defects to membrane components, and modifications in energy and redox metabolism [27]. Having in mind that middle storage represents a key time-point for the above-mentioned RBC insults, it came as no surprise that this is when all proteasome activities reach their maximum membrane levels. Indeed, ROS [20] and calcium accumulation, carbonylation of membrane proteins, lipid peroxidation [22], as well as the rewiring of the metabolism [28] occur mainly between the second and the third week of storage in CPD-SAGM. In G6PD-deficient RBCs, proteasome subunits seem to co-translocate to the membrane, hand in hand with a variety of members of the “Repair or Destroy” proteins [18], including antioxidant enzymes and chaperones. In a similar way, the 20S core particle (total and activity) interactomes (Figure 4, Figure 5, Figure 6 and Figure 7) exhibited numerous linkages with heat shock proteins, peroxiredoxins, T-complex components, etc.

### 4.2. Time-Dependent Cross-Talk between Proteasome Activities and ROS Levels

The repeatable correlation between the proteasome activities and the intracellular ROS further supports the responsiveness of the 20S proteasome toward oxidative-driven defects [29]. It is tempting to hypothesize that ROS levels positively correlate with proteasome activity until the middle of the storage period because the proteasome is still capable of counteracting the accelerated storage-driven oxidative stress. However, since the machinery itself is also a target of oxidation [30], leading to inactivation or other functional defects, it cannot probably cope with the overwhelming redox imbalance of RBCs at the end of storage, resulting in the inverse correlation profile evident in the cytosol of βThal^+^ RBCs and the membrane of control RBCs. The previously detected accumulation of inactive proteasomes in the supernatant of blood units in late-storage [19] reinforces this claim.

The discrete correlation profile between ROS and proteasome activities in the cytosol for βThal^+^ RBCs and the membrane for control RBCs might be indicative of (a) a higher need for protein control in the cytosol of βThal^+^ RBCs, since their membrane seems to be more “protected” (as evidenced by the minor levels of lipid peroxidation or protein carbonylation [22] and the excess of membrane-bound chaperones and redox enzymes [23]), or (b) a different (likely more complex) regulation of membrane proteostasis in βThal^+^ RBCs, which is not dependent on variations in ROS levels in the cytosol. This finding probably reflects the redox behavior of βThal^+^ RBCs as imprinted upon the metabolism, proteome, and resistance to storage lesion. After all, proteasome activity can be regulated by many factors, including post-translational modifications of subunits, such as phosphorylation [31]. Indeed, the discrete expression of kinases and phosphatases in the membrane of βThal^+^ RBCs [23] and their higher connectivity with the proteasome activity in the respective biological networks (Figure 6 and Figure 7) when compared to the controls further support this more complex proteostatis regulation.

### 4.3. Storage Induces Higher Levels of Proteasome Activity in the Membrane of βThal^+^ RBCs

Despite having baseline levels and a storage time-course profile similar to the control, CASP-like and CH-like activities presented higher values in the membrane of βThal^+^ from mid-storage onwards. In the same context, EVs released from the βThal^+^ membrane—where abundant working proteasomes are attached—contain more active proteasome subunits. This is in line with recent proteomic studies showing a higher abundance of several proteasome subunits and molecular chaperones, which is known to preserve the proteolytic activity of the proteasome [32], in the membrane of stored βThal^+^ RBCs versus control [23], as well as in old or sickle cell disease RBCs in vivo [33]. The same trend for high proteasome activity has been reported in G6PD-deficient stored RBCs [18], which is another distinct genetic group characterized by sustained levels of oxidative stress. Consequently, the elevated proteasome activity in the membrane could represent an adaptation of RBCs to redox imbalance in vivo [29], which might be recalled by them to better preserve the proteome stability and overall functionality when exposed to additional oxidative challenges, including those imposed by the storage-induced accelerated aging. Although the specific activity of the proteasome in the membrane of RBCs has not been studied so far, high proteasome activities have been reported in the ROS-enriched cytosol of RBCs from sickle cell disease patients [34], which further increased following treatment with hydroxycarbamide [35]. Interestingly, even though βThal^+^ RBCs are less susceptible to storage hemolysis [22], the βThal^+^ supernatant was characterized by higher proteasome activity. Previous studies have shown that release of hemoglobin and 20S proteasome particles happen in a parallel manner in the supernatant of control RBC units, though the activity of free proteasomes reaches a plateau upon day 28 [19]. It is possible for the activity to be better preserved in βThal^+^ RBCs either through a more effective molecular chaperoning of the 20S subunits [23,32] or due to their physical endurance of the sustained oxidative stress they experience in vivo. It should not be omitted that within beta-thalassemic precursor cells, proteasome subunits are upregulated in order to decongest the α-globin-filled cells [36].

### 4.4. Biological Networking of Proteasome and Proteasome Activities

During maturation, RBCs lose their organelles along with their ability to synthesize proteins. Moreover, upon refrigerated storage, the RBCs undergo an accelerated aging process characterized by redox imbalance, metabolic rewiring, and physiological dysfunctions, including proteotoxic stress. Taken together, these facts highlight the important role of proteostasis in the RBC life cycle. Goodman et al. [10] have shown that the proteostasis machinery is localized in the “heart” of the RBC interactome, as evidenced by protein–protein interaction analysis and further confirmed by larger-scale pathway and network analyses [37]. Our bioinformatic analysis validates and expands these findings by showing the same core position not only of proteasome subunits but also of their individual activities in RBC interactomes enriched with several omics and physiological data. However, it has been suggested that proteasome pools of different subcellular location, subunit composition, and activities may exert distinct functions in cell homeostasis [38]. Generally considered as cytoplasmic in location, the currently reported biological networks highlight several associations of 20S membrane activity with parameters never shown before in RBCs. The numerous correlations with cytosolic components of the 20S and 19S particles, molecular chaperones, ubiquitinylation and redox enzymes, and the proteolytic system of calpain/calpastatin in both groups signify the membrane activity of proteasome as part of a membrane proteostasis macromolecular system in stored RBCs that is subjected to a concerted spatiotemporal regulation: it might be membrane-bound en block. It seems that in the absence of lysosomes, the RBC proteasomes team up with the cytosolic calpain system to regulate degradative processes at the membrane level during storage. Co-variations of proteasome and calpain activities have been reported in several stressful or pathological conditions [39]. Nonetheless, βThal^+^ stored RBCs seem to possess a more complex proteostatic network compared to controls, given the fact that their early-storage interactome involves almost 25% more connections between the 20S core particles and peroxiredoxins, HSPs, T-complex components, etc. The complete loss of this difference until the end of the storage period supports the above-mentioned hypothesis that βThal^+^ RBCs are “primed” to cope with additional levels of oxidative, energy, and proteotoxic stresses, such as the ones that come hand in hand with storage conditions. Similar results have emerged from interactome analyses in sickle cell RBCs [10].

The activity of membrane proteasomes also co-varied with the levels of membrane lipids and proteins in situ. Co-variation with proteins participating in lipid composition, such as the fatty acid synthase, suggests interactions at either structural (e.g., lipid composition of the membrane domain) or regulatory levels. Similar cross-talks have been detected in other cells and animal models. The N-myristoylation-driven membrane localization of the proteasome controls its phosphorylation [40], and in turn, the proteasome activity controls the abundance and activity of enzymes participating in lipid biosynthesis and metabolism [41,42].

Apart from lipids per se, several membrane proteins exhibited negative correlations with the membrane expression and activity of proteasomes, especially at late storage, when the membrane was disorganized by accumulated storage lesion. Several of them (e.g., 4.1R, glycophorin C, myosins, CD44) were components of the updated “repair or destroy” box of the RBC interactome [37]. Of particular importance were the raft-resident flotillins, which are currently correlated not only with the membrane levels of 20S proteins but also with their activity in βThal^+^ membranes, suggesting a regulatory role for the lateral compartmentalization of the membrane, similar to the one reported in LPS-stimulated macrophages [43]. Of note, fatty acid synthase is also recruited to the macrophage lipid rafts upon LPS treatment [43]. Moreover, the proteasome-enriched membrane of sickle cell disease RBCs has been characterized by decreased levels of RBC raft-resident flotillins and stomatin [10].

The βThal^+^ proteasome networks did not only differ from controls in terms of degree of connectivity but also in their specificity. Heterozygotes’ proteostasis is intertwined with a great variety of βThal^+^ specific membrane proteome features in stored RBCs [22,23], producing an interesting subnetwork that seems to characterize these donors. For instance, the connections of proteasome activities with piezo-1 and Na^+^/K^+^ ATPase molecules might prove to be of great importance, since earlier studies have implicated proteasome pathways in modulating the surface expression of several ion channels [44]. In this context, the improved resistance of stored βThal^+^ RBCs to mechanical stress could be partly related to the higher proteasome maintenance and activity in the membrane and the lower levels of piezo-1. Indeed, it has been found that mechanical stretch triggers piezo-1 degradation via the proteasome in endothelial cells [45]. Another molecule that is more abundant in βThal^+^ membranes is arginase-1, which is known to compete nitric oxide synthase for their common substrate L-arginine. Its eclectic presence in the βThal^+^ networks might be indirect, since it is known that the proteasome’s activity is related to the function of endothelial nitric oxide synthase [46]. Finally, the observed dialogue between energy metabolism and the proteasome has already been hinted at in the past, in both RBCs [10,37] and nucleated cells. Interestingly, in the latter ones, energy homeostasis seems to be partly regulated by a ubiquitin-independent proteasome system [47]. The pre-storage differences (increase in glycolysis) observed in the energy metabolism of βThal^+^ RBCs [22] and the higher expression of both metabolic enzymes and proteasome subunits on their stored membranes [23] might be the reason behind the higher connectivity between energy-related proteins and the proteasome in this group.

## 5. Conclusions

Membrane proteostasis in stored RBCs is based not only on cytosolic but also on membrane regulatory mechanisms, including proteasomes, working in situ. Intracellular and extracellular proteasome activities vary as a function of the cytosolic ROS levels, the storage age of RBCs, and the presence of βThal^+^ mutations. Storage is associated with decreased proteasome activity in the cytosol but increased activity in the membrane and the extracellular supernatant. Furthermore, while β-globin mutations in the heterozygous state do not seem to impact the proteasome activity in vivo, storage stress promotes the elevation of proteasome activity in the membrane of βThal^+^ RBCs. According to the interactome analyses, variation in membrane activity in stored cells seems to be connected to the lipid composition, the lateral compartmentalization, the structural disorganization, and the binding of the “repair-or-destroy” group of proteins. The highly enriched 20S interactome of stored βThal^+^ RBCs is indicative of an impressive and primed “proteo-vigilance”, which is likely related to their redox metabolism and distinct membrane protein profile. In the light of recent findings showing the modulation of old-stored RBCs’ proteasome activities by transfusion-mimicking conditions in vitro [18], along with evidence for physiologically important functions of proteasome-containing platelet-derived EVs [48], the currently reported data deserve further investigation in potential RBC transfusion settings in vivo, including patients treated with proteasome inhibitors.

## Figures and Tables

**Table 1:** Time course evaluation of proteasome activity during red blood cell storage stratified by topology.

		Days of Storage				
	Activities	7	14	21	28	42
Cytosol	CH-like	34 ± 8	29 ± 7 *	24 ± 5 *	24 ± 6 *^,§^	21 ± 5 *^,§^
	CASP-like	21 ± 5	18 ± 5 *	15 ± 5 *^,§^	14 ± 4 *^,§^	14 ± 4 *^,§^
	TR-like	44 ± 12	40 ± 10	31 ± 10 *^,§^	29 ± 8 *^,§^	30 ± 11 *^,§^
Membrane	CH-like	42 ± 19	48 ± 21	55 ± 21	46 ± 21	39 ± 20 ^#^
	CASP-like	40 ± 13	52 ± 16	81 ± 35 *^,§^	81 ± 29 *^,§^	63 ± 34 ^#^
	TR-like	29 ± 11	34 ± 9	50 ± 22 *^,§^	36 ± 18 ^#^	29 ± 14 ^#^
Supernatant	CH-like	0.3 ± 0.1	0.3 ± 0.2	0.5 ± 0.3	0.5 ± 0.3	0.7 ± 0.3 *^,#^
	CASP-like	1.3 ± 0.5	1.7 ± 0.7	1.8 ± 0.5 *	1.7 ± 0.7 *	1.9 ± 0.6 *
	TR-like	3.4 ± 1.0	3.6 ± 0.8	3.9 ± 1.2	4.0 ± 0.9 *^,#^	4.5 ± 1.3 *

CH: chymotrypsin, CASP: caspase, TR: trypsin; (*) *p* < 0.05 vs. day 7, (^§^) *p* < 0.05 vs. day 14, (^#^) *p* < 0.05 vs. day 21. Data are shown as mean ± SD in ×10^3^ RFU/mg of protein.

## Data Availability

The proteomics data used in this study are available online at https://www.mdpi.com/1422-0067/22/7/3369 (accessed on 25 March 2021). All physiological data presented in this study are available upon request.

## Supplementary Materials

The following are available online at https://www.mdpi.com/article/10.3390/membranes11090716/s1. Table S1. Numerical code used for the presentation of proteomic and physiological parameters in the biological networks shown in Figure 3, Figure 4, Figure 5, Figure 6 and Figure 7.
