# Supplementary material for: Red Blood Cell Proteasome in Beta-Thalassemia Trait: Topology of Activity and Networking in Blood Bank Conditions
Source: Membranes (Basel). 2021 Sep 17;11(9):716. doi: 10.3390/membranes11090716 (PMC8466122; doi:10.3390/membranes11090716)
Supplement: Supplementary file 1 [file membranes-11-00716-s001.zip › membranes-1339961-supplementary.pdf]

Supplementary Materials: Red Blood Cell Proteasome in Beta-thalassemia Trait: Topology of Activity and Networking in Blood Bank Conditions

**Alkmini T. Anastasiadi [1,†], Vassilis L. Tzounakas [1,†], Vasiliki-Zoi Arvaniti [1], Monika Dzieciatkowska [2],
Konstantinos Stamoulis [3], Marilena E. Lekka [4,*], Issidora S. Papassideri [1], Angelo D'Alessandro [2],
Anastasios G. Kriebardis [5] and Marianna H. Antonelou [1,*]**

[1] Department of Biology, School of Science, National and Kapodistrian University of Athens (NKUA), 15784 Athens, Greece; alkanast@biol.uoa.gr (A.T.A.); tzounak@biol.uoa.gr (V.L.T.); vazoarvaniti@gmail.com (V.-Z.A.); ipapasid@biol.uoa.gr (I.S.P.)
[2] Department of Biochemistry and Molecular Genetics, School of Medicine–Anschutz Medical Campus, University of Colorado, Aurora, CO 80045, USA; monika.dzieciatkowska@ucdenver.edu (M.D.); ANGELO.DALESSANDRO@UCDENVER.EDU (A.D.)
[3] Hellenic National Blood Transfusion Centre, Acharnes, 13677 Athens, Greece; kostas.stamoulis@gmail.com
[4] Laboratory of Biochemistry, Department of Chemistry, University of Ioannina, 45110 Ioannina, Greece
[5] Laboratory of Reliability and Quality Control in Laboratory Hematology (HemQcR), Department of Biomedical Sciences, School of Health & Welfare Sciences, University of West Attica (UniWA), 12243 Egaleo, Greece; akrieb@uniwa.gr
* Correspondence: manton@biol.uoa.gr (M.H.A.); mlekka@uoi.gr (M.E.L.)
† These authors contributed equally to this work.

Table S1. Numerical code used for the presentation of proteomic and physiological parameters in the biological networks shown in Figures 3–7.

Parameter	Abbreviation
14-3-3 protein epsilon	1
2',3'-cyclic-nucleotide 3'-phosphodiesterase	2
26S proteasome non-ATPase regulatory subunit 1	3
26S proteasome non-ATPase regulatory subunit 11	4
26S proteasome non-ATPase regulatory subunit 12	5
26S proteasome non-ATPase regulatory subunit 13	6
26S proteasome non-ATPase regulatory subunit 14	7
26S proteasome non-ATPase regulatory subunit 2	8
26S proteasome non-ATPase regulatory subunit 3	9
26S proteasome non-ATPase regulatory subunit 5	10
26S proteasome non-ATPase regulatory subunit 6	11
26S proteasome non-ATPase regulatory subunit 7	12
26S proteasome non-ATPase regulatory subunit 8	13
26S proteasome regulatory subunit 10B	14

26S proteasome regulatory subunit 4	15
26S proteasome regulatory subunit 6A	16
26S proteasome regulatory subunit 6B	17
26S proteasome regulatory subunit 7	18
26S proteasome regulatory subunit 8	19
55 kDa erythrocyte membrane protein	20
Acetylcholinesterase	21
Actin, alpha skeletal muscle	22
Actin, cytoplasmic 1	23
Acylamino-acid-releasing enzyme	24
Aldehyde dehydrogenase family 16 member A1	25
Alpha-adducin	26
Alpha-centractin	27
Ammonium transporter Rh type A	28
Ankyrin-1	29
Annexin A4	30
Annexin A7	31
AP-1 complex subunit beta-1	32
AP-2 complex subunit alpha-1	33
AP-2 complex subunit alpha-2	34
AP-2 complex subunit beta	35
AP-2 complex subunit mu	36
Aquaporin-1	37
Arginase-1	38
Atlastin-3	39
ATP-binding cassette sub-family B member 6	40
ATP-binding cassette sub-family G member 2	41
ATP-citrate synthase	42
ATP-dependent 6-phosphofructokinase	43
ATP-dependent 6-phosphofructokinase	44
Band 3 anion transport protein	45
Basal cell adhesion molecule	46
Basigin	47
Beta-2-glycoprotein 1	48
Beta-actin-like protein 2	49
Beta-adducin	50
Blood group Rh(CE) polypeptide	51
BMP-2-inducible protein kinase	52
C-1-tetrahydrofolate synthase, cytoplasmic	53
Calmodulin-1	54
Calnexin	55
Calpain small subunit 1	56
Calpain-1 catalytic subunit	57
Calpain-5	58
Calpastatin	59
Calreticulin	60

cAMP-dependent protein kinase catalytic subunit alpha 61
cAMP-dependent protein kinase type I-alpha regulatory subunit 62
Carbonic anhydrase 1 63
Casein kinase I isoform alpha 64
Caspase-like activity (cytosol) 65
Caspase-like activity (membrane) 66
Caspase-like activity (supernatant) 67
Catalase 68
CB1 cannabinoid receptor-interacting protein 1 69
CD44 antigen 70
CD59 glycoprotein 71
Cell division control protein 42 homolog 72
Chymotrypsin-like activity (cytosol) 73
Chymotrypsin-like activity (membrane) 74
Chymotrypsin-like activity (supernatant) 75
Clathrin heavy chain 1 76
Coiled-coil and C2 domain-containing protein 1A 77
Complement decay-accelerating factor 78
Complement receptor type 1 79
COP9 signalosome complex subunit 2 80
COP9 signalosome complex subunit 3 81
COP9 signalosome complex subunit 4 82
COP9 signalosome complex subunit 6 83
Copine-3 84
Copper-transporting ATPase 1 85
Cullin-1 86
Cullin-associated NEDD8-dissociated protein 1 87
Delta-aminolevulinic acid dehydratase 88
Dematin 89
Deoxyribose-phosphate aldolase 90
Diamide-induced ROS generation 91
Disheveled-associated activator of morphogenesis 1 92
DnaJ homolog subfamily B member 1 93
DnaJ homolog subfamily C member 13 94
Dynactin subunit 1 95
Dynactin subunit 2 96
Dynamin-2 97
E2 ubiquitin-conjugating enzyme 98
E3 ubiquitin-protein ligase HUWE1 99
E3 ubiquitin-protein ligase UBR4 100
Elongation factor 1-alpha 101
Endonuclease domain-containing 1 protein 102
Endoplasmic reticulum chaperone BiP 103
Endoplasmic reticulum resident protein 44 104
Endoplasmin 105
Epoxide hydrolase 1 106

Equilibrative nucleoside transporter 1	107
Erythrocyte band 7 integral membrane protein	108
Erythrocyte membrane protein band 4.2	109
Erythroid membrane-associated protein	110
Exportin-7	111
Extracellular potassium levels	112
Extracellular sodium levels	113
Extracellular vesicles' procoagulant activity	114
Ezrin	115
F-actin-capping protein subunit alpha-1	116
F-actin-capping protein subunit beta	117
Fatty acid synthase	118
Flavin reductase (NADPH)	119
Flotillin-1	120
Flotillin-2	121
Fructose-bisphosphate aldolase A	122
Fructose-bisphosphate aldolase C	123
G protein-coupled receptor kinase 6	124
Galectin-3	125
Gamma-adducin	126
Glutathione S-transferase LANCL1	127
Glyceraldehyde-3-phosphate dehydrogenase	128
Glycophorin-A	129
Glycophorin-C	130
GMP reductase 1	131
Golgi-associated plant pathogenesis-related protein 1	132
GTPase KRas	133
GTPase NRas	134
GTPase-activating protein and VPS9 domain-containing protein 1	135
Guanine nucleotide-binding protein G(i) subunit alpha-2	136
Guanine nucleotide-binding protein G(I)/G(S)/G(T) subunit beta-1	137
Guanine nucleotide-binding protein G(I)/G(S)/G(T) subunit beta-2	138
Guanine nucleotide-binding protein G(k) subunit alpha	139
Guanine nucleotide-binding protein G(q) subunit alpha	140
Guanine nucleotide-binding protein G(s) subunit alpha isoforms short	141
Guanine nucleotide-binding protein subunit alpha-13	142
Heat shock 70 kDa protein 1A	143
Heat shock 70 kDa protein 4	144
Heat shock cognate 71 kDa protein	145
Heat shock protein HSP 90-alpha	146
Heat shock protein HSP 90-beta	147
Hematocrit	148
Hemoglobin subunit alpha	149
Hemoglobin subunit beta	150
Hemoglobin subunit delta	151

Hsc70-interacting protein	152
Immunoglobulin gamma-1 heavy chain	153
Immunoglobulin heavy constant gamma 2	154
Immunoglobulin heavy constant gamma 3	155
Immunoglobulin heavy constant mu	156
Immunoglobulin kappa constant	157
Immunoglobulin kappa light chain	158
Immunoglobulin lambda-like polypeptide 5	159
Importin subunit beta-1	160
Importin-5	161
Importin-7	162
Intercellular adhesion molecule 4	163
Intermediate conductance calcium-activated potassium channel protein 4	164
Intracellular calcium levels	165
Intracellular hemoglobin	166
Intrinsic ROS generation	167
Irrevesible RBC shape transformation (percentage)	168
Junctional adhesion molecule A	169
Kell blood group glycoprotein	170
Keratin, type I cytoskeletal 10	171
Keratin, type I cytoskeletal 9	172
Keratin, type II cytoskeletal 1	173
Keratin, type II cytoskeletal 2	174
Keratin, type II cytoskeletal 6A	175
LanC-like protein 2	176
L-lactate dehydrogenase B chain	177
Long-chain-fatty-acid--CoA ligase 3	178
Long-chain-fatty-acid--CoA ligase 4	179
Long-chain-fatty-acid--CoA ligase 6	180
Lymphocyte function-associated antigen 3	181
Mean Cell Volume (MCV)	182
Mean Corpuscular Fragility (MCF)	183
Mean Corpuscular Hemoglobin (MCH)	184
Mean Corpuscular Hemoglobin Concentration (MCHC)	185
Mechanical Fragility	186
Membrane Lipid Peroxidation	187
Methyltransferase-like protein 7A	188
Moesin	189
Monocarboxylate transporter 1	190
Multidrug resistance-associated protein 1	191
Multidrug resistance-associated protein 4	192
Multidrug resistance-associated protein 5	193
Multiple inositol polyphosphate phosphatase 1	194
Myosin light chain 4	195
Myosin regulatory light chain 12B	196

Myosin-10	197
Myosin-9	198
NADH-cytochrome b5 reductase 3	199
Neuropathy target esterase	200
Neutral alpha-glucosidase AB	201
Neutral cholesterol ester hydrolase 1	202
Nucleosome assembly protein 1-like 1	203
Nucleosome assembly protein 1-like 4	204
Oxidative hemolysis	205
Peptidyl-prolyl cis-trans isomerase B	206
Peptidyl-prolyl cis-trans isomerase FKBP3	207
Peroxiredoxin-1	208
Peroxiredoxin-2	209
Phenylhydrazine-induced ROS generation	210
Phosphatidylinositide phosphatase SAC1	211
Phosphatidylinositol 4-kinase type 2-alpha	212
Phosphatidylinositol 5-phosphate 4-kinase type-2 alpha	213
Phosphatidylinositol-binding clathrin assembly protein	214
Phosphatidylserine exposure	215
Phospholipid scramblase 1	216
Phospholipid transfer protein C2CD2L	217
Phosphoribosyl pyrophosphate synthase-associated protein 2	218
Piezo-type mechanosensitive ion channel component 1	219
Plasma membrane calcium-transporting ATPase 1	220
Plasma membrane calcium-transporting ATPase 4	221
Polypyrimidine tract-binding protein 1	222
Polyubiquitin-B	223
Probable ubiquitin carboxyl-terminal hydrolase FAF-X	224
Proteasome activator complex subunit 1	225
Proteasome activator complex subunit 2	226
Proteasome adapter and scaffold protein ECM29	227
Proteasome subunit alpha type-1	228
Proteasome subunit alpha type-2	229
Proteasome subunit alpha type-3	230
Proteasome subunit alpha type-4	231
Proteasome subunit alpha type-5	232
Proteasome subunit alpha type-6	233
Proteasome subunit alpha type-7	234
Proteasome subunit beta type-1	235
Proteasome subunit beta type-2	236
Proteasome subunit beta type-3	237
Proteasome subunit beta type-4	238
Proteasome subunit beta type-5	239
Proteasome subunit beta type-7	240
Protein 4.1	241
Protein arginine N-methyltransferase 5	242

Protein argonaute-2 243
Protein carbonylation 244
Protein DDI1 homolog 2 245
Protein diaphanous homolog 1 246
Protein disulfide-isomerase 247
Protein disulfide-isomerase A3 248
Protein disulfide-isomerase A6 249
Protein XRP2 250
Pyrroline-5-carboxylate reductase 3 251
Radixin 252
Ras-related C3 botulinum toxin substrate 1 253
Ras-related protein Rab-10 254
Ras-related protein Rab-18 255
Ras-related protein Rab-21 256
Ras-related protein Rab-2B 257
Ras-related protein Rab-35 258
Ras-related protein Rab-5C 259
Ras-related protein Rab-7a 260
Ras-related protein Rab-8A 261
Ras-related protein Rab-8B 262
Ras-related protein Ral-A 263
Ras-related protein Rap-1A 264
Ras-related protein Rap-1b 265
Ras-related protein Rap-2a 266
Ras-related protein Rap-2b 267
Red blood cell count (RBC) 268
Red cell distribution width (RDW) 269
Retinal dehydrogenase 1 270
Ribose-phosphate pyrophosphokinase 1 271
RuvB-like 1 272
RuvB-like 2 273
Semaphorin-7A 274
Serum albumin 275
SH3 domain-binding glutamic acid-rich-like protein 276
SH3 domain-binding glutamic acid-rich-like protein 2 277
Small integral membrane protein 1 278
Small membrane A-kinase anchor protein 279
Sodium/potassium-transporting ATPase subunit alpha-1 280
Solute carrier family 2, facilitated glucose transporter member 1 281
Solute carrier family 40 member 1 282
Sorbitol dehydrogenase 283
Spectrin alpha chain, erythrocytic 1 284
Spectrin beta chain, erythrocytic 285
Storage hemolysis 286
Stress-induced-phosphoprotein 1 287
Syntaxin-7 288

Syntaxin-binding protein 3	289
TBC1 domain family member 24	290
T-complex protein 1 subunit alpha	291
T-complex protein 1 subunit beta	292
T-complex protein 1 subunit delta	293
T-complex protein 1 subunit epsilon	294
T-complex protein 1 subunit eta	295
T-complex protein 1 subunit gamma	296
T-complex protein 1 subunit theta	297
T-complex protein 1 subunit zeta	298
Tensin-1	299
tert-butyl-hydroperoxide induced ROS generation	300
Total antioxidant capacity (supernatant)	301
Transforming protein RhoA	302
Transitional endoplasmic reticulum ATPase	303
Transmembrane emp24 domain-containing protein 2	304
Transmembrane protein 222	305
Tripeptidyl-peptidase 2	306
Tropomodulin-1	307
Tropomyosin alpha-1 chain	308
Tropomyosin alpha-3 chain	309
Trypsin-like activity (cytosol)	310
Trypsin-like activity (membrane)	311
Trypsin-like activity (supernatant)	312
Ubiquitin carboxyl-terminal hydrolase 14	313
Ubiquitin carboxyl-terminal hydrolase 15	314
Ubiquitin carboxyl-terminal hydrolase 5	315
UDP-glucose:glycoprotein glucosyltransferase 1	316
Unconventional myosin-XVIIIa	317
Urea transporter 1	318
Uric acid dependent antioxidant capacity (supernatant)	319
Uric acid independent antioxidant capacity (supernatant)	320
Vacuolar protein sorting-associated protein 13A	321
Very-long-chain 3-oxoacyl-CoA reductase	322
Vesicle-associated membrane protein 3	323
Vesicle-associated membrane protein-associated protein A	324
Vesicle-fusing ATPase	325
Vesicle-trafficking protein SEC22b	326
Vesicular integral-membrane protein VIP36	327
V-type proton ATPase subunit B	328
WD repeat-containing protein 81	329
WD repeat-containing protein 91	330
Zinc transporter 1	331
